# CDC Grand Rounds: Newborn Screening for Hearing Loss and Critical Congenital Heart Disease

**DOI:** 10.15585/mmwr.mm6633a4

**Published:** 2017-08-25

**Authors:** Scott D. Grosse, Tiffany Riehle-Colarusso, Marcus Gaffney, Craig A. Mason, Stuart K. Shapira, Marci K. Sontag, Kim Van Naarden Braun, John Iskander

**Affiliations:** ^1^National Center on Birth Defects and Developmental Disabilities, CDC; ^2^University of Maine College of Education and Human Development, Orono, Maine; ^3^University of Colorado School of Public Health, Aurora, Colorado; ^4^New Jersey Department of Health; ^5^Office of the Associate Director for Science, CDC.

Newborn screening is a public health program that benefits 4 million U.S. infants every year by enabling early detection of serious conditions, thus affording the opportunity for timely intervention to optimize outcomes (*1*). States and other U.S. jurisdictions decide whether and how to regulate newborn screening practices. Most newborn screening is done through laboratory analyses of dried bloodspot specimens collected from newborns. Point-of-care newborn screening is typically performed before discharge from the birthing facility. The Recommended Uniform Screening Panel includes two point-of-care conditions for newborn screening: hearing loss and critical congenital heart disease (CCHD). The objectives of point-of-care screening for these two conditions are early identification and intervention to improve neurodevelopment, most notably language and related skills among infants with permanent hearing loss, and to prevent death or severe disability resulting from delayed diagnosis of CCHD. Universal screening for hearing loss using otoacoustic emissions or automated auditory brainstem response was endorsed by the Joint Committee on Infant Hearing in 2000 and 2007* and was incorporated in the first Recommended Uniform Screening Panel in 2005. Screening for CCHD using pulse oximetry was recommended by the Advisory Committee on Heritable Disorders in Newborns and Children in 2010 based on an evidence review^†^ and was added to the Recommended Uniform Screening Panel in 2011.^§^

## Universal Screening for Hearing Loss

Permanent hearing loss present at birth affects approximately 1.6 of every 1,000 infants in the United States (*2*). Early hearing detection and intervention (EHDI) programs at the state and federal levels promote a “1-3-6” plan that includes 1) screening all infants at age ≤1 month, 2) performing diagnostic audiologic evaluation of infants who do not pass screening at age ≤3 months, and 3) providing appropriate intervention for children with diagnosed hearing loss at age ≤6 months. Children with permanent hearing loss who receive intervention services before age 3–6 months have significantly better language development than do children who do not receive services (*3*). Similarly, early diagnosis of hearing loss, starting with newborn screening, has been shown to reduce deficits in receptive and expressive language that occur in unscreened children who subsequently receive a clinical diagnosis of hearing loss (*4*).

Universal newborn hearing screening also can yield long-term economic benefits (*5*). A prospective British cohort study that tracked groups of children with permanent bilateral hearing loss who were either screened soon after birth or later in infancy found that at school age, children in the newborn screening cohort had significantly better receptive language and substantially lower educational costs (*6*). Extrapolating from those data, a U.S. study estimated potential averted special education costs of approximately $200 million per year, which would largely offset the cost of hearing screening (*7*).

Statewide newborn hearing screening programs began to be established in the 1990s. By the early 2000s, all states had established publicly funded EHDI programs that provide 1) technical assistance to providers, 2) support for families, and 3) data tracking to ensure receipt of services in accordance with the 1-3-6 goals. State EHDI programs receive technical assistance and funding from CDC or the Health Resources and Services Administration (HRSA). CDC provides funding to states to develop and implement data systems that help ensure that infants receive recommended screening, diagnosis, and intervention services. CDC also conducts an annual survey to assess progress toward achieving EHDI goals. In addition, CDC supports evaluation and research on long-term clinical outcomes and program effectiveness. HRSA provides funding and technical assistance to states to support quality improvement activities, family engagement, and activities to reduce loss to follow-up of infants who do not pass the newborn hearing screening.

National EHDI data^¶^ have demonstrated improvements in the number of infants meeting the 1-3-6 goals. From 2000 to 2014, the percentage of newborns who had documented newborn hearing screening increased from 52% to >97%, and the number of documented diagnoses of hearing loss following screening increased sixfold, from 855 in 2000 to 6,163 in 2014. Although almost all U.S. infants now undergo hearing screening soon after birth, infants who fail to pass screening do not necessarily receive timely diagnostic evaluations or timely intervention services once they receive a diagnosis of permanent hearing loss. Therefore, EHDI programs focus on increasing the percentage of infants who meet the 3-month diagnostic evaluation and 6-month early intervention goals. Since 2005, states have reported aggregated data through the CDC Hearing Screening and Follow-up Survey on the numbers of infants successfully receiving those recommended services (*2*). From 2005 to 2014, the percentage of infants who failed newborn hearing screening and who were documented by their state EHDI program as having received a completed diagnostic evaluation increased from 30% to 58%. Among infants with confirmation of hearing loss, documented enrollment in early intervention during the same period increased from 58% to 65%. There have also been reductions in the number of infants lost to follow-up/lost to documentation (failure to report the results from hearing screening, rescreening, diagnostic services, or treatment services to the state EHDI program and the medical home), both overall and in selected states (*2*). For example, just 4.6% of infants who did not pass newborn hearing screening in Massachusetts in 2014 were lost to follow-up/lost to documentation, and 85% of infants with diagnosed hearing loss were documented to have received intervention services.

Further progress in the timely provision of newborn hearing screening, diagnostic, and intervention services as well as improved standardization of data are possible through state-based EHDI Information Systems (EHDI-IS). These EHDI-IS support the early identification of hearing loss and receipt of intervention by enabling state programs to document and track those infants not passing the newborn hearing screening and in need of follow-up services. CDC provides technical assistance and funding to maintain and strengthen these systems. To improve the completeness and accuracy of reported data, CDC collaborated with state EHDI programs to develop a set of Functional Standards for EHDI-IS.** These standards specify technical and functional requirements for EHDI-IS and list data items considered important for tracking and surveillance by EHDI programs.

## Screening for Critical Congenital Heart Disease

CCHD includes 12 structural heart disorders that prevent the heart from pumping blood normally to the body, resulting in a high likelihood of low blood oxygen saturation.^††^ CCHD screening relies on noninvasive pulse oximetry; diagnosis of CCHD requires evaluation by a specialist. CCHD occurs in approximately two of every 1000 births.^§§^ Infants with undetected CCHD who are discharged from a birth hospital are at risk for developing serious complications that could result in emergency readmission or death within the first few days or weeks of life. Although many cases of CCHD are detected prenatally or through clinical examination, infants who appear normal might be discharged home and subsequently undergo life-threatening crises. It has been estimated that before the introduction of newborn screening for CCHD, 70–100 infants died each year in the United States from late-diagnosed CCHD (*8*). The cost of pulse oximetry screening is estimated to be $10–$15 per infant (*9*). Using conservative estimates of averted deaths and hospitalization costs, an economic analysis calculated that CCHD screening appears cost-effective relative to other services (*10*).

Newborn screening for CCHD has been implemented more recently than newborn hearing screening; the first state policies were adopted in 2011 (*11*). As of 2016, 48 states had laws or policies on CCHD screening. In contrast to long-established EHDI programs, CCHD screening programs are in the early stages of development, and no federal funding is available to support state CCHD screening activities. There is no national collection or analysis of CCHD screening data, and among states, data collection procedures differ. A HRSA-funded newborn screening technical assistance center has built a data repository and begun to collect information from states on newborns with CCHD who were identified by screening and had not received a prenatal or clinical diagnosis.

Many states have birth defects surveillance programs that collect information on children with various types of major birth defects, and some states with birth defects surveillance programs might have the capability to evaluate effectiveness of CCHD screening (*12*). New Jersey was the first state to implement mandatory statewide CCHD screening in all its birthing facilities on August 31, 2011 (*13*). One day after the requirement to screen all infants was implemented, a baby who did not pass CCHD screening was determined to have CCHD and underwent life-saving surgery with a successful outcome. Upon implementation in 2011, New Jersey assessed screening coverage through aggregate quarterly reports from all birthing facilities and collected information on all failed screens through a CCHD screening module built into the New Jersey Birth Defects Registry (NJBDR). The module captures clinical information needed to evaluate the unique contribution of screening to early identification of CCHD. Data from this module are reviewed monthly by NJBDR staff members, and follow-up with hospitals for clarification is conducted as needed. Confirmed records are then entered into a separate NJBDR analytic database. New Jersey now collects individual-level CCHD screening data on all live births through its electronic birth certificate and continues to ascertain detailed information on infants who fail the screen through its CCHD module in the NJBDR. New Jersey’s high rate of screening coverage and successful ongoing use of the NJBDR have been achieved through employment of extensive education and training efforts. The key to the success of the New Jersey CCHD screening program has been collaboration among the NJBDR, hospitals, community partners, and the Office of Vital Statistics and Registry. More complete reporting of CCHD screening on all infants and linkage with birth defects surveillance systems are important for ensuring that all infants are screened, optimizing the screening algorithm and quantifying the contribution to improved health (*12*).

Universal CCHD screening continues to evolve. States have implemented various screening algorithms that are being evaluated for specific settings, such as births at high elevation or infants in neonatal intensive care units. Evaluation of the impact of CCHD screening on deaths from CCHD is also under way, using administrative data found in national linked infant birth and death records. Improved data collection will be crucial to assess the effectiveness and guide optimization of CCHD screening (*14*).

## Challenges and New Directions in Point-of-Care Newborn Screening in the United States

EHDI is a mature point-of-care screening program that has demonstrated health and economic benefits. Lessons learned from EHDI can be applied to both CCHD screening and point-of-care newborn screening for other conditions that might be included in the Recommended Uniform Screening Panel in the future. The interface between public health and hospitals, health care providers, and families in point-of-care screening presents both challenges and opportunities across conditions (*15*). As demonstrated by EHDI, the data tracking and follow-up capacity of public health agencies can facilitate early identification of affected infants and ongoing coordination between families and clinical care systems. By promoting screening, timely diagnosis, and follow-up based on standardized data systems, public health workers and agencies can play critical roles in enabling children with permanent hearing loss or CCHD to be healthy and reach their full potential.

## Footnotes

* http://www.asha.org/policy/PS2007-00281/.

† https://www.hrsa.gov/advisorycommittees/mchbadvisory/heritabledisorders/nominatecondition/reviews/cyanoticheart.pdf.

§ https://www.hrsa.gov/advisorycommittees/mchbadvisory/heritabledisorders/recommendations/correspondence/cyanoticheartsecre09212011.pdf.

†† https://www.cdc.gov/ncbddd/heartdefects/hcp.html.

§§ https://ghr.nlm.nih.gov/condition/critical-congenital-heart-disease.pdf.
